# A randomised controlled trial of the Nextdoor Kind Challenge: a study protocol

**DOI:** 10.1186/s12889-021-11489-y

**Published:** 2021-08-05

**Authors:** Michelle H. Lim, Pamela Qualter, Alexandra Hennessey, Ben J. Smith, Taylah Argent, Julianne Holt-Lunstad

**Affiliations:** 1 Iverson Health Innovation Research Institute, Swinburne University of Technology, Hawthorn, Victoria Australia; 2 Manchester Institute of Education, University of Manchester, Manchester, UK; 3 University of Sydney, Camperdown, NSW Australia; 4 Brigham Young University, Provo, UT USA

**Keywords:** Community interventions, Physical health, Mental health, Randomised controlled trial

## Abstract

**Background:**

Community interventions are often promoted as a way of reducing loneliness and social isolation in our neighbourhoods. However, those community interventions are rarely examined within rigorous study designs. One strategy that holds the potential to reduce loneliness and can promote health and wellbeing is doing acts of kindness. The current study involves evaluating the impact of kindness acts on loneliness in community-dwelling individuals using an online social networking platform.

**Methods:**

This study is made up of three randomised controlled trials conducted in three countries. Each randomised controlled trial has two arms (intervention vs waitlist control) and is designed to compare the effectiveness of the KIND challenge, which involves doing at least one act of kindness per week within a four-week period. This study will recruit users of an online community, be randomised online, and will be conducted using online assessments. We will first explore the effects of the intervention on the primary outcome of loneliness, followed by secondary outcomes, social isolation, neighbour relationship quality and contact, mental health symptoms, stress, quality of life, and positive affect. Further, we will assess the feasibility, acceptability, and safety of the KIND Challenge.

**Discussion:**

This study, designed to evaluate the impact of kindness on the community, will be the first large scale randomised control trial conducted across three countries, Australia, UK, and USA. It will examine the potential of community-led interventions to reduce loneliness, improve social isolation, and promote neighbourhood cohesion, health, and wellbeing, which is especially crucial during the COVID-19 public health crisis.

**Trial registration:**

Clinical Trials Registry. NCT04398472. Registered 21st May 2020.

**Supplementary Information:**

The online version contains supplementary material available at 10.1186/s12889-021-11489-y.

## Background

The health effects of social isolation are well known to be detrimental to health outcomes [1, 2] and more recently, the evidence for perceived social isolation, “loneliness”, on poor health outcomes, has also been growing [3, 4]. Those who lack strong social connections show a 50% increased risk of an earlier death, and when we consider loneliness specifically, there is a 26% increase in early death after taking into consideration factors like age, gender, and ethnicity [5]. Prolonged loneliness experienced over the course of childhood and adolescence is associated with poor health behaviours and poorer mental health during early adulthood [6, 7]. There are a range of physical health indicators associated with loneliness. For example, cardiovascular health indicators including systolic blood pressure, heart rate, heart contractibility, and cardiac output are all impaired in lonely people [2, 8–10].

The negative impacts of loneliness on health outcomes are seen across the lifespan [11]. While there may be differences in the prevalence and source of loneliness at different ages [11], our communities are one place where people of all ages can take an opportunity to connect with others [12], building connections with neighbours and attaining a sense of belonging. Being well connected in a community setting can provide an important source of social interaction and can mitigate against social isolation and feelings of loneliness [13]. According to the belongingness hypothesis, individuals need a sense of connection to larger groups or a community as well as close interpersonal relationships to achieve a sense of belonging. In a survey of 2840 people in the UK, the variable most consistently associated with having higher subjective wellbeing was ‘feeling part of a community’ [14]. Community connectedness can be increased and doing so can bring about great social and personal benefits to members of the community [14]. This is commonly achieved by ensuring accessibility and equity for all groups in the community. For example, local councils may improve access to home and community care services for people from diverse backgrounds and with diverse needs and run events such as community festivals that promote community connectedness [15]. Those methods rely on the active participation of community members, and despite best efforts, there are challenges faced in engaging those who are isolated and most in need of these opportunities.

Previously, a sense of community would have been established through face-to-face contact, but the use of social networking and other forms of social technology now offers an alternative method of connecting and engaging members within a community. In comparison to face-to-face interactions, programs that involve web-based communication can bypass location related restrictions and allow people to extend their relationships with the wider community. However, it has been found that having access to social networking may not lead to less loneliness [16, 17]. This is consistent with the loneliness paradox [18] where we may be more connected than ever digitally, but not have meaningful connections that satisfy our social needs. This is not to say that digital technology is ineffective in mitigating loneliness, but what is needed is the implementation of evidence-based strategies that can be implemented or promoted within these digital platforms. One example of an online platform that has the potential to address social isolation and loneliness is Nextdoor Inc., a social networking service for neighbourhoods. The Nextdoor online platform, based in 11 countries, including the UK, Australia, and the USA, offers community members a social network opportunity and can use the platform to promote evidence-based strategies to increase the quality of social relationships (in this case neighbourhood ties). There is, therefore, opportunity to evaluate and test any evidence-based strategies aimed at improving the meaningfulness of social ties and reducing loneliness, in addition to increasing the number of social ties (i.e., reducing social isolation).

One strategy that has already shown preliminary feasibility within an online platform [19] to improve relationship ties within a community is performing acts of kindness towards others. During the COVID-19 public health crisis, communities have shown an increase in community spirit by sharing their experiences, showing solidarity, and offering help [20, 21]. There is evidence in smaller studies that acts of kindness can indeed promote wellbeing [19, 22] and improve relationship quality [23, 24]. Acts of kindness are often integrated within strengths-based positive psychology interventions which are used to increase positive affect and improve wellbeing [25, 26]. In a sample of socially anxious adults aged 29–70 years, those who performed three acts of kindness a day for 2 days each week showed greater satisfaction in their relationships with acquaintances, co-workers, friends, and close friends (*t* (40) = 2.25, *p* < 0.05, *d* = 0.35). In comparison to the other experimental conditions commonly used in this population (i.e., identifying safety behaviours, and documenting life events), performing acts of kindness was the only condition shown to be significant in increasing participant’s satisfaction with their social relationships. Therefore, engaging in acts of kindness appears to increase relational functioning [23]. In another study, kindness and gratitude-based positive psychology activities (PPA) were used to elicit positive social interactions with peers and improve relationship satisfaction [24]. In a randomised controlled trial (RCT), 225 participants aged 18 to 66 years were assigned to one of three conditions (relationship-focused, self-focused or control). Participants reported on relationship satisfaction, social support, and happiness across three time points (baseline, post-intervention and six-week follow up). The experimental PPAs included (1) relationship-focused (involving either doing gratitude or kindness activity) or (2) self-focused (no social interaction). Those who completed relationship- focused PPAs reported greater increases in relationship satisfaction compared with those in the self-focused and active control condition at follow-up. Additionally, those in the relationship-focused condition reporting feeling their existing friendships had improved at the end of the intervention and effects remained at follow up. Taken together, PPAs that promote kindness and gratitude can improve relationship satisfaction.

In terms of reducing loneliness, there is also emerging evidence that positive psychology interventions that include doing acts of kindness hold the potential to reduce loneliness (amongst other benefits, including improving psychological wellbeing, positive affect, mental health) in people with and without mental health disorders [27–29]. But what remains unknown is the impact of acts of kindness within larger scale community interventions, particularly when assessed outside a larger toolbox of positive psychology strategies (e.g., increasing positive affect). While acts of kindness are often promoted, the effects of those are poorly understood and rarely examined within rigorous study designs. For example, do acts of kindness improve community ties (e.g., neighbours)? And if so, what is the dose response and duration require? In other words, how many acts of kindness does one have to carry out (and over what duration) before we detect an increase in relationship quality and improvements in health and wellbeing? Answers to those questions are largely unknown because there has been little focus on the evaluation of community interventions.

### Objectives

The overall objective is to examine the social and psychological impact of kindness on a community sample. This will be examined by asking community members within an online social networking platform, Nextdoor, to participate in performing at least four acts of kindness over a four-week period. We refer to this as the Kindness Is Nextdoor (KIND) challenge.

The primary intervention objective of the Nextdoor KIND Challenge is to reduce loneliness in the Nextdoor online communities across three countries (Australia, United States, United Kingdom). We expect significant changes to loneliness. Specifically, it is anticipated that challenge participants will show significantly greater improvement in loneliness compared with the control group, after accounting for known covariates.

A secondary intervention objective of the Nextdoor KIND Challenge is to reduce social isolation risk in the Nextdoor online communities across three countries (Australia, United States, United Kingdom). It is anticipated that challenge participants will show significantly greater reduction in social isolation when compared with the control group.

An additional secondary intervention objective of the KIND Challenge is to improve neighbourhood relationship quality and contact, and we expect significant improvements in neighbourhood ties both in terms of relationship quality and contact. It is anticipated that KIND Challenge participants, compared with the control group, will show greater improvement in neighbourhood relationship quality as measured by social cohesion and trust, perception that their neighbourhood is improving, higher importance around getting to know their neighbours, greater positivity and lower negativity towards their neighbors, and lower neighbourhood conflict. It is anticipated that KIND Challenge participants compared with the control group will also report significantly more neighbourhood contacts compared with the control group.

There are further secondary objectives of the KIND Challenge to improve mental health outcomes, that is lower depression, social anxiety, and stress levels, improve quality of life, and increase positive affect, with those participants in the KIND challenge showing greater positive effects when compared with control participants.

We also anticipate that acts of kindness will be feasible, acceptable, and safe to do for community participants (see apriori in Implementation secondary outcomes section).

- Hypothesis 1 (H1): There will be a reduction in loneliness in participants assigned to the Nextdoor KIND Challenge groups compared to the waitlist control group post the 4-week intervention.
- Hypothesis 2 (H2): There will be a reduction in social isolation in participants assigned to the Nextdoor KIND Challenge groups compared to the waitlist control group post the 4-week intervention.
- Hypothesis 3 (H3): There will be an improvement in neighbourhood relationship quality and contact outcomes (a) social cohesion and trust, (b) perception that their neighbourhood is improving, (c) higher importance around getting to know their neighbours, (d) more likely to be classed as having a supportive neighbour network than not, (e) have lower neighbourhood conflict, and (f) increased number of neighbourhood contacts, in participants assigned to the Nextdoor KIND Challenge groups compared to the waitlist control group post the 4-week intervention.
- Hypothesis 4 (H4): There will be an improvement in mental health secondary outcomes, lower (a) depression and (b) social anxiety, (c) stress, and greater (d) quality of life, and (e) positive affect, in participants assigned to the Nextdoor KIND Challenge groups compared to the waitlist control group post the 4-week intervention.

While it is vital to investigate the impact of KIND Challenge post intervention, it is possible for impact on outcomes to be distal; that is indirect effects caused by changes in proximal outcomes. The KIND Challenge aims to reduce loneliness and social isolation, which may lead to later distal impact on other outcomes. Capturing impact at one-month follow-up after the completion of the KIND Challenge enables modelling the maintenance of intervention effects, i.e., are effects sustained at one-month or are sleeper effects present? In addition to understanding whether effects of the intervention are sustained, it is also important to know whether the intervention itself is sustainable. The KIND Challenge acts are positive, engaging, and feasible to the average individuals, and thus designed to be sustainable.

- Hypothesis 5 (H5): The Nextdoor KIND Challenge effects noted in H1–4 above will be maintained at one-month follow-up for the participants randomly allocated to KIND Challenge group.
- Hypothesis 6 (H6): The KIND Challenge is sustainable, participants assigned to the KIND Challenge will continue engaging in acts of kindness at one-month follow-up.

The third objective of this intervention is to examine factors that may moderate the effects, including dosage, pre-existing levels of social connection, type of kindness activity, and adherence. Interventions are rarely adhered to in full, and variability in compliance and engagement is commonplace; moreover, this variability is associated with differences in expected outcomes. It is therefore important to take measures of engagement with the intervention to explore how variability in engagement moderate outcomes. First, it is anticipated that the level of adherence (do participants complete the instructed four acts of kindness) and engagement, or *dosage*, in terms of frequency and duration of KIND activity will make a difference to loneliness and social isolation severity. Second, the more connected participants feel in relation to the KIND activity will impact outcomes and participants are likely to feel less lonely. Third, it is recognized that some acts of kindness may have greater effect than others, and specifically how do different acts of kindness that provide either emotional, tangible, informational, belonging, or companionship support impact on loneliness and social isolation will be examined. Therefore, both quantity and quality of engagement with the KIND challenge can be modelled as predictors of primary outcomes of loneliness and social isolation. Finally, understanding engagement and adherence to the intervention is fundamental to exploring the feasibility and making recommendations for future interventions. Therefore, an additional purpose of the study is to identify reasons, (i.e., lack of time, illness, work, stress etc.) for lack of engagement and adherence to the KIND challenge. In terms of analysis, traditional approaches to dealing with (non) compliance in RCTs (e.g., ‘as treated’) produce biased estimates, so we will use Complier Average Causal Effect (CACE) estimation to explore the impact of compliance to implementing the intervention. CACE allows exploration of treatment effects where there is less than ideal levels of participant compliance (as opposed to the offer of said intervention, as in ITT used for H1 above).

- Hypothesis 7 (H7): The impact of the Nextdoor KIND Challenge on the primary outcome of loneliness and secondary outcomes of social isolation, neighbourhood relationship quality and contact, neighbourhood connectedness, and mental health will vary as a function of intervention adherence and engagement. Specifically, we would predict larger effect sizes for participants who (a) adhered and engaged more in the KIND Challenge and (b) feel more connected to their neighbours, and (c) we would predict that levels of engagement with different acts of kindness, i.e., emotional support, tangible support, informational support, belonging support, and companionship support to have different effects.
- Hypothesis 8 (H8): The magnitude of intervention effects noted in H1–4 above will vary as a function of intervention compliance. Specifically, we predict larger effect sizes for participants classified as compliers in terms of number of kind acts completed (dosage).

The final objective of this intervention is to explore additional factors that may influence participants’ experiences or the effectiveness of the intervention. These relate to (1) how loneliness and social isolation differ across countries, (2) how loneliness and social isolation differ across neighbourhood characteristics (urban vs rural), and demographic variables (age, gender, socioeconomics), (3) how COVID-19 impacts loneliness and social isolation, and on people’s interactions with their neighbours. Consistent with the evaluation of community-based interventions, the role of demographic and particular neighbourhood characteristics should be incorporated as potential covariates in the analyses.

## Methods

### Trial design

**Fig. 1:**
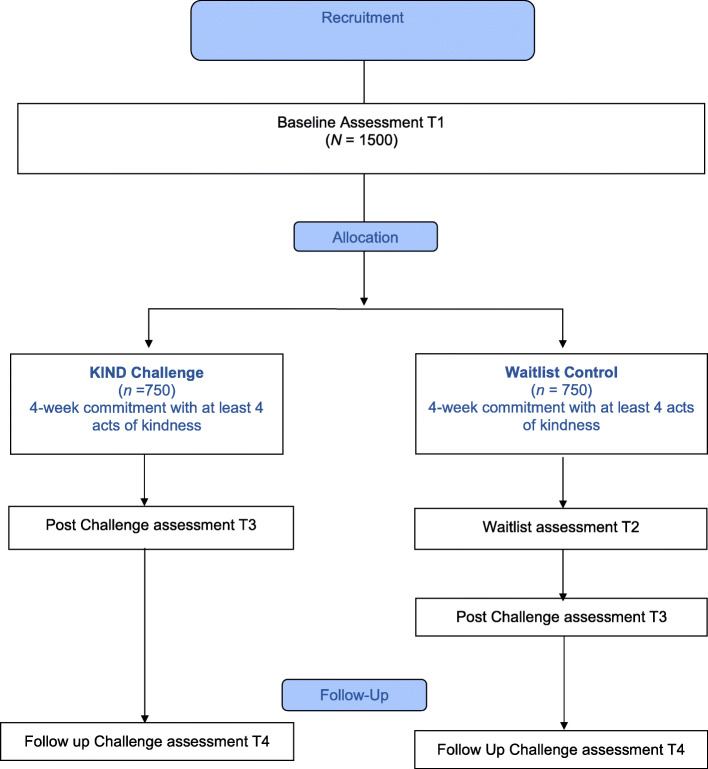
CONSORT diagram showing trial design for each country, UA, UK and Australia

This study is made up of three RCTs and each RCT has two conditions: the KIND Challenge condition and a waitlist control condition. A person enrolled into the KIND Challenge condition will be asked to do acts of kindness at least four times for a duration of 4 weeks and will complete three online assessments. A person enrolled into a waitlist control condition will wait 4 weeks before starting the KIND Challenge and will be required to complete four online assessments. The evaluation of the KIND Challenge will be pre-registered prior to the study launch. Please refer to Fig. 1.

### Study setting

This trial will be conducted online in three countries (Australia, United States of America, and the United Kingdom). All measures will be administered through the online survey platform Qualtrics.

### Eligibility criteria

The inclusion criteria will reflect the real-world characteristics of community dwellers who report a need to connect. This includes those aged 18 to 90 years, who are registered Nextdoor users who are willing to commit to the challenge for a specific period of time. Exclusion criteria includes individuals without proficient English reading comprehension skills.

### Who will take informed consent?

Participants are all over 18 years old and can provide informed consent online.

### Intervention – Nextdoor KIND challenge

For those receiving the KIND challenge, they will be asked to nominate different activities they could do over a four-week period to address loneliness in their communities. Because the KIND challenge takes place during the COVID-19 pandemic, participants will be asked to adhere to their state or country’s health department safety guidelines when undertaking these activities. We note here the different types of support that can be selected. Participants are also provided an opportunity to specify their activity in a free text box if none of the presented options were relevant.

- Emotional support. Show care and concern for a neighbour (e.g., provide a listening ear, cheer up a neighbour who is down, check in on the welfare of a neighbour)
- Informational support. Provide advice or helpful information to a neighbour (e.g., where to shop, good doctors in the area, potential job opportunities, gardening tips, etc.)
- Tangible support. Help a neighbour out (e.g., mow their lawn, take in their garbage bins, offer meals, run errands, bring them groceries, etc.)
- Belonging support. Contribute to a larger neighbourhood effort, action, or activity (e.g., support a neighbourhood business, share your talents/skills with others, neighbourhood clean-up, volunteering, etc.)
- Companionship support. Have regular contact with a neighbour (e.g., chat across the fence, street or balcony, call on the phone, etc.)
- Other. Please specify what you did in the textbox.

The KIND Challenge activities have been selected because they are considered to be positive, engaging, and feasible to the average individual, and draw upon the social support literature to tap into emotional support, informational support, tangible support, belonging support [30], and companionship [31–33]. Participants who are enrolled in the Challenge condition will receive an email reminder to complete the activity weekly till the next post-Challenge assessment. After the four-week period, participants in the KIND Challenge condition will be emailed a link to the post challenge survey. One month after this, they will be emailed a link to the final follow up survey. For those in the KIND Challenge group, there will be three surveys over a two-month period if the participant follows the intended structure of the project.

### Waitlist condition

It will be clearly outlined to participants that the trial involves a waitlist control prior to enrolment, and informed consent will include agreement to undergo a 4-week waitlist period and an additional assessment prior to commencing the KIND Challenge. Waitlist participants will be instructed to continue with their usual activities throughout the waitlist period. Waitlist participants received one email 2 weeks prior to remind them of their upcoming enrolment into the Challenge condition.

### Outcomes

**Fig. 2:**
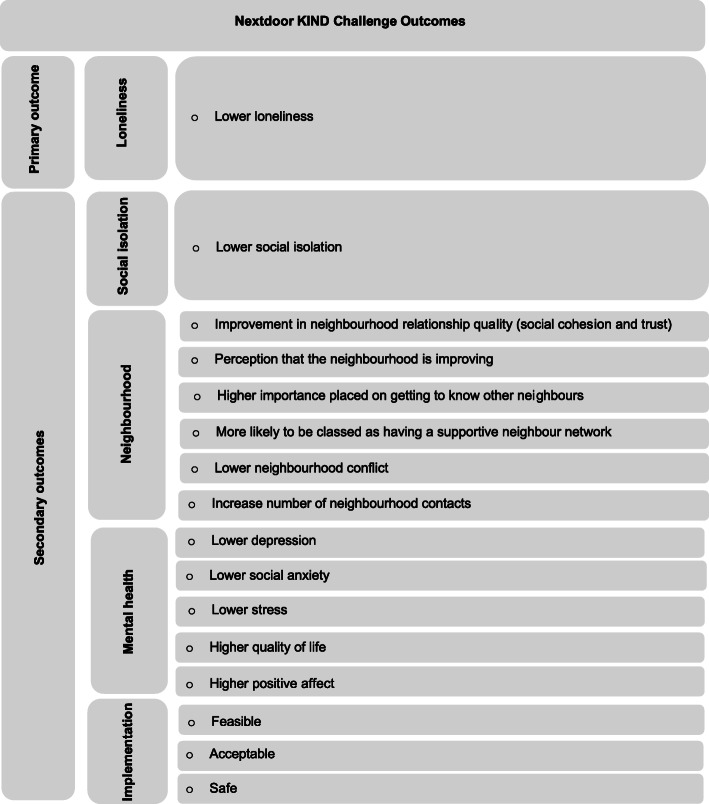
Nextdoor KIND Challege primary (i.e proximal) and secondary (i.e distal) outcomes

Information about a participant’s demographics, mental health, stress, affect, quality of life, and their perception of the level of community engagement and neighbourhood activity within their own neighbourhood will be collected. The questionnaires are standardised tools that measure physical health, mental health, stress, affect, quality of life, and community engagement. We will also include ratings related to behaviour change. This includes questions to measure the actions selected to interact with others, frequency, and duration of those actions (i.e., dose response), and meaningfulness of those actions. See Table 1 for measures and when they are assessed. We further categorised the outcomes into three sections: intervention primary outcome, intervention secondary outcomes, and implementation secondary outcomes (see Fig. 2).

**Table 1:** SPIRIT schedule of enrolment, intervention, and assessments of the Nextdoor KIND Challenge

Time point	Baseline T1	Waitlist T2^a^	Post Intervention T3	1 Month Follow Up T4
Enrolment				
Informed consent	X			
Allocation	X			
Assessments				
Demographic form	X			
UCLA-LS3	X	X	X	X
LSNS-18	X	X	X	X
EUROHIS-QOL-8	X	X	X	X
PHQ-8	X	X	X	X
Mini-SPIN	X	X	X	X
PSS-4	X	X	X	X
PANAS	X	X	X	X
CE	X			
SCS	X	X	X	X
Number of Contacts	X	X	X	X
Neighbourhood Perception of Change	X	X	X	X
Neighbourhood Importance	X	X	X	X
Neighbourhood Modified Social Relationship Index	X	X	X	X
Neighbourhood Conflict	X	X	X	X
Neighbourhood interaction since COVID-19	X	X	X	X
Pre-KIND Challenge Neighbourhood Contact and Previous Kindness activity	X	X		
Nextdoor Platform Activity	X	X	X	X
COVID-19 Social Restrictions	X	X	X	X
Challenge Activity Nomination	X^b^	X		
Post-KIND Challenge Activity			X	
Safety			X	
Post-Kind Challenge Overall Impact			X	
Follow-Up KIND Challenge Activity				X

Note. *UCLA-LS3* refers to UCLA Loneliness - Scale Version 3, *LSNS* refers to Lubben Social Network Scale, *EUROHIS-QOL-8* refers to European Health Interview Survey Quality of Life-8, *PHQ 8* refers to Patient Health Questionnaire-8, *Mini-SPIN* refers to Mini-Social Phobia Inventory, *PSS-4* refers to Perceived Stress Scale-4, *PANAS* refers to Positive and Negative Affect Scale, *CE* refers to Community Engagement, *SCS refers to* Social Capital Scale

^a^Only the waitlist group completed T2

^b^Only those randomised into the Challenge condition will be asked to nominate Challenge activities

### Intervention primary outcome

#### Loneliness

The UCLA Loneliness Scale - Version 3 (UCLA-LS; [34]) is a 20-item measure employing a 1 (Never) to 4 (Always) ordinal scale. The measure consists of both positively- and negatively- worded items that assess loneliness (e.g., How often do you feel that you are no longer close to anyone?). The UCLA-LS has been shown to correlate negatively with life satisfaction and perceived social support, thus supporting its convergent validity with related constructs.

### Intervention secondary outcomes

#### Social isolation

The Lubben Social Network Scale (LSNS: [35]) is an 18-item scale that assesses the frequency and quality of contact – such as talking about private matters – in an individual’s network. There are three subscales, and each consists of 6 items relating to family, neighbour and friend connections. The scale employs a 0 (none) to 5 (nine or more) ordinal scale and includes 6 items (e.g., How many relatives do you see or hear from at least once a month?). Higher scores indicate lower risk of social isolation. The scale has demonstrated adequate levels of reliability and the proposed clinical cut-points showed good convergent validity [35].

#### Neighbourhood relationship quality and contact

Neighbourhood relationship quality specific variables that we anticipate would be influenced by the KIND Challenge include neighbourhood social cohesion and trust, perception of change, importance, ambivalence, and conflict. Additionally, the number of neighbourhood contact may also increase because of the KIND Challenge.

#### Neighbourhood social cohesion and trust as measured by social capital scale [36]

The Social Capital Scale is a 7-item measure designed to assess social cohesion and trust in a community. The scale was derived from a similar scale used in Sampson et al. [37]. This measure consists of both positively and negatively worded items and is recorded on a 2-point dichotomous format ranging from 0 (strongly disagree or disagree) to 1 (strongly agree or agree). This measure was shown to be a reliable tool to measure social cohesion and trust at a neighbourhood level [37].

#### Neighbourhood perception of change [38]

This question used by a previous study by Bateman and colleagues [38], asks if participants thought their neighbourhood was improving from 1 (improving), 2 (stable) or 3 (declining).

#### Neighbourhood importance [38]

This question used by a previous study by Bateman and colleagues [38], asks how important participants thought it was to know their neighbours from *1 (very important) to 5 (very important)*.

#### Social relationship index - Neighbourhood modified [39, 40]

The 3-item scale was modified from the Social Relationship Index (SRI) which measures both positivity and negativity in relationship ties which can indicate if one has supportive, ambivalent, or aversive ties [39, 40]. We ask participants how helpful, upsetting, and mixed/conflicted their feelings are towards their neighbours when needing advice, understanding, or a favour – rated on a Likert scale from 1 (not at all) to 6 (extremely). Based on these scores and using a threshold model classification, participants are then categorised to either having a supportive, aversive, or ambivalent neighbour relationship.

#### Neighbourhood conflict

Participants will be asked to reflect on their interactions with their neighbours in the past month using a dichotomous yes/no to the following: absence/presence of neighbour conflict, critical comments towards the participant, participant critical of neighbours. Responses will be dichotomously scored as either reporting no instances of neighbourhood contact or reporting at least one instance of neighbourhood conflict. These questions were developed for the study (see Supplementary Table 1 – reference a).

#### Neighbourhood number of contacts

This item asks the number of people the participants know in their neighbourhood, 0 (0–5 neighbours) to 4 (15+ neighbours). This question was developed for the study (see Supplementary Table 1 reference b).

#### Depression

The Patient Health Questionnaire-8 (PHQ-8; [41]) is an 8-item measure of depression severity based on 8 of the 9 item criteria of the DSM-IV. The measure was derived from the PHQ-9 however; the ninth question was omitted from this study because of ethics reasons around asking about suicidality. Depression severity is scored based on the presence of depressive symptomology in the previous 2 weeks of measure admission. The scale uses a 1 (not at all) to 4 (nearly every day) ordinal scale with higher scores indicating higher levels of depressive symptomology. The PHQ-9 has been shown to have good criterion and construct validity and excellent internal consistency [42]. The PHQ-8 shares similar properties in terms of validity and reliability and is therefore an adequate alternative to its 9-item scale counterpart [41].

#### Social anxiety

The Mini-Social Phobia Inventory (Mini-SPIN; [43]) is a brief 3-item measure of generalised social anxiety disorder. The measure employs a 5-point ordinal scale form 1 (not at all) to 5 (extremely), with higher scores indicating a greater level of generalised social anxiety. The Mini-SPIN has shown high sensitivity in detecting social anxiety disorder [43].

#### Quality of life

The European Health Interview Survey-Quality of Life – 8-Item Index (EUROHIS-QOL-8; [44]) is an 8-item measure of quality of life consisting of questions that assess overall QOL, general health, energy, daily living activity, self-esteem, social relationships, finances, and home. The measure is derived from the WHOQOL-BREF and shares a similar 5-point Likert-scale response format. The scale has demonstrated good qualities in term of internal consistency (Cronbach’s alpha = 0.83) and satisfactory convergent and discriminant validity [44].

#### Positive affect

The Positive and Negative Affect Schedule – SF (PANAS-PA 10 item; [45]). is a 10-item positive affect subscale measure of the PANAS scale. The measure asks participants to rate the extent to which they feel a particular emotion along a 5-point Likert scale ranging from 1 (very slightly) to 5 (extremely). This subscale measure has been shown to have high levels of internal consistency [45] and acceptable levels of convergent validity [46].

#### Stress

The Perceived Stress Scale-4 (PSS-4; [47]) is a four-item measure of stress was derived from the original 14-item Perceived Stress Scale (PSS). This measure consists of both positively and negatively worded items that asses an individual’s evaluation of stressful events. The measure employs a 5-point ordinal scale ranging from 0 (never) to 4 (very often). The PSS-4 has been shown to negatively correlate with levels of perceived health, social support, being male, and older age [48]. The scale has demonstrated fair reliability and adequate psychometric properties [48].

### Implementation secondary outcomes

#### Feasibility

Feasibility will be the following: having a high retention rate (i.e., < 40% drop-out).

#### Acceptability

We measure acceptability around these factors, including how connected (1 not at all connected to 10 very connected), how meaningful was the KIND Challenge activity (1 not very meaningful to 10 very meaningful), how safe they felt when completing the KIND Challenge (1 not very safe to 10 very safe), how positive they felt (1 not very positive to 10 very positive), how comfortable they felt doing the KIND Challenge activity (1 not at all comfortable to 10 very comfortable). It is anticipated that the Nextdoor KIND Challenge will yield ratings of more than 5 indicating higher levels of acceptability across these outcomes. These questions were specifically developed for the study (see Supplementary Table 1 section c).

#### Safety

We assess for unintended harms (e.g., conflict) during this period. In this case, we measure the occurrence of neighbor conflict because of the KIND Challenge. It is anticipated that the KIND Challenge activity is safe for participants with no unintended conflicts reported. This question was developed for the study (see Supplementary Table 1 section d).

#### Post-KIND challenge activity

*Post intervention,* participants will be asked if they completed either their nominated KIND Challenge activity or any KIND Challenge activity over the past month. Type of support can be categorized based on participant selection. Participants can also select “other” activities that were not listed. If the “other” box activity is selected, participants are asked to describe the activity in detail via a free text entry box. Responses in the free text box entry will be coded independently by two research assistants not related to this trial. Inter-rater reliability between coders will be calculated to ensure adequate reliability of categorization. For each activity, participants will be asked how frequently they completed the activity (1 = once to 4 = more than 5 times), the duration of the activity (1 = less than 10 min to 5 = more than 2 h), how connected to others they felt after completing the selected activity (1 = not at all connected to 10 = very connected). They can select more than one activity and will rate each activity according to those scales. If participants have not completed a KIND Challenge activity, an additional multiple-choice question will be included to assess for what reasons the activity could not be completed (e.g., lack of time, that they forgot, illness, work, stress etc). Participants were then asked to reflect on the KIND Challenge overall including how meaningful and safe they found the challenge, how positive they felt after the challenge, and how connected and comfortable they felt towards their neighbours because of the challenge. These questions were specifically developed for the study (see Supplementary Table 1 - section c).

#### Post-kind challenge overall impact

The measure includes 10 items pertaining to the effects of the Nextdoor KIND Challenge. Six items (looking forward to being with neighbours; more connected to neighbours; more socially confident; form new friendships; more connected to community; more positive feelings will be assessed using a Likert scale ranging from 1 (extremely disagree) to 5 (extremely agree) with higher responses reflecting greater success of the KIND Challenge. The remaining 4 items were recorded in an open-ended format allowing participants to explain what can help them feel more connected, what being connected means to them, what prevented participants from connecting and to provide feedback about how the KIND Challenge was ran. These questions were specifically developed for the study (see Supplementary Table 1 – section e).

#### Follow up KIND challenge activity

At 1 month follow-up**,** participants will be asked if they have been in contact with their neighbours in the past month (Yes/No). Participants are asked if they have *continued* with the Kindness activity, if so, which activity or activities they chose to continue. Participants will be asked how frequently they completed the activity (1 = once to 4 = more than 5 times), the duration (1 = less than 10 min to 5 = more than 2 h), and how connected they felt to others after completing the activity (1 = not at all to 10 = very). If they had not continued a Challenge activity, participants will be asked to report the main reasons for not continuing with the KIND Challenge activity in a textbox provided. These questions were specifically developed for the study (see Supplementary Table 1 – section f).

### Adjusting for potential covariates

Several variables may need to be adjusted for in analyses examining the impact of the KIND Challenge and related objectives. This includes factors known to influence our primary outcomes such as: baseline community engagement, current Nextdoor platform activity, neighbour interactions since COVID-19, other COVID-19 social restrictions including level of social restrictions, and changes to face-to face interactions. Further adjustments may be needed to account for factors that may influence participation in the Challenge. These variables include measures of previous neighbour contact, and previous acts of kindness and the uptake of activities has to be accounted for, and pre-Challenge activities are measured. Post-Kind Challenge activity from number of activities take up, frequency, duration and impact of these activities are measured. An overall impact is also assessed and to understand if benefits of the KIND Challenge are sustained, we ask participants the same questions in relation to neighbour contact and the KIND Challenge activity uptake, frequency, duration, and impact.

#### Community engagement [49]

Questions were designed to assess an individual’s involvement in cultural and community-based groups/activities in the past year. The measure consists of two subscales with 2 and 7 items respectively and assesses the frequency with which an individual attends cultural and community events, groups, or activities. The measure employs a 3-point ordinal scale ranging from 0 (less than once a year) to 2 (every few months or more). Both “community cultural engagement” and “community group engagement” subscales have been shown by Fancourt et al. [49] to have good reliability and internal consistency (cultural engagement Cronbach’s alpha = 0.74, group engagement alpha = 0.69).

#### Nextdoor platform activity

A list of questions were developed to determine how participants currently engage with the Nextdoor platform. The measure consists of up to 9 items which include which will ask how long a user has been a member of Nextdoor ranging from 1 (less than 1 week) to 6 (more than 5 years), how many active and passive hours the participant has spent on the platform in the past month on a scale of 1 (less than 1 h) to 4 (more than 9 h). The last item relates to a change in Nextdoor platform activity due to the COVID-19 pandemic using a dichotomous yes/no response. If yes, participants will be asked if they have spent more or less time on the Nextdoor platform. These questions were specifically developed for the study (see Supplementary Table 1 – section g).

#### Neighbour interactions since COVID-19

Questions were developed to evaluate how the COVID-19 pandemic has influenced interactions between neighbours. The participants will be asked about their current contact with their neighbours, specifically if overall frequency of contact has changed from 1 (no change), 2 (in contact with neighbours more) or 3, (in contact with neighbours less). If participants select that they have been in contact with their neighbours more, they are asked about the contact modality (e.g., in person, at a safe distance, via phone text, videocall or other communication device etc.). We will also assess participant’s worries about their health, other people’s health, interaction avoidance, additional methods of communication to get in touch, meaningfulness of the relationship rated on a 1 (no worries/ not done/ strongly disagree) to 10 (worry a lot/ done this a lot/ strongly agree) Likert scale. These questions were specifically developed for the study (see Supplementary Table 1 – section h).

#### COVID-19 social restrictions

This measure was designed to assess the impact of the COVID-19 pandemic on immediate social interactions. Social restriction severity will be measured by the number of different government restrictions relevant to the participant at time of survey (from a list of 13 types of restrictions). This list consists of a variety of imposed restrictions including social, travel, retail, and education. These questions were specifically developed for the study (see Supplementary Table 1 – section i).

#### Pre-KIND challenge neighbourhood contact and previous kindness activity

Prior neighbourhood contact may influence both the primary outcome and participation in the Challenge. Thus, participants will be asked if they have been in contact with their neighbour safely in person or using a digital communication device (Yes/No). They are then asked if they had completed an act of kindness before being assigned to the Challenge. If yes, participants will be asked how frequently they completed the activity (1 = once to 4 = more than 5 times), the duration of the activity (1 = less than 10 min to 5 = more than 2 h) and how connected they feel to their neighbours because of the activity (1 = not at all connected to 10 = very connected). These questions were specifically developed for the study (see Supplementary Table 1 – section j).

### Sample size

Because there are no large-scale community-led randomised controlled trial interventions with reducing loneliness as a primary outcome, we had to estimate power based on the number of participants and maximum number potential covariates we will need to account for. Power calculations were calculated using PowerUp! [50]. Power was set at 0.80 and an Alpha threshold of 0.05. Estimated pre-post correlations for loneliness are between .35 and .83 [51, 52] and for a conservative estimate the lowest correlations for each outcome are used. Therefore, for a projected sample of 1500 participants per country and with up to 20 co-variates for the primary outcomes of loneliness, a minimum detectable effect size (MDES) for a trial effect is .14 in an intention-to-treat (ITT) analysis. Given recent recommendations that Cohen’s effect size benchmarks are too stringent to detect important effects in social and personality psychology [53, 54], we see an effect size of .14 being of small-medium size that is of some explanatory and practical use, even if only in the short-term and therefore clinically meaningful. Further, noting that the strength of an intervention is best evaluated in relation to outcomes it aims to achieve [55], we are guided by a meta-analysis of loneliness intervention trials that found a mean intervention effect size of − 0.198, but with much variability (95% CI: − 0.32, − 0.08; [56]). Thus, previous research has typically found small-medium effects on average for loneliness interventions and this study is sufficiently powered to detect effects of a comparable magnitude.

### Recruitment and enrolment

We will recruit through a sample of Nextdoor users within each country using online promotions and, or an email to all users within each country database. The recruitment strategy will not be constrained to neighbourhood location or demographics in order to ensure that we recruit a diverse range of participants in order to generalise the data across the different Nextdoor communities. We will aim to recruit approximately 1500 community dwellers aged 18 to 90 years across each country (Australia, UK, USA), with an overall estimate of 4500 participants across three countries. These recruitment targets are feasible given the number of users active within each country (at least 500,000 users).

The external organisation Nextdoor will be responsible for recruitment of participants and will invite users within each country without specific parameters. This is to ensure we recruit all kinds of users, including those who are high and low users of the program, and those who are from low to high socio-economic status. Potential participants will respond to advertisements embedded in the Nextdoor website platform that they are already a member of. Nextdoor will recruit via email, push notifications (website and app). If an individual is interested in participating, they will be directed to a Qualtrics survey containing a participant information statement and consent form. This Qualtrics survey is only accessible by Swinburne University of Technology. In this survey, participants will provide online consent to take part in Nextdoor KIND Challenge. Following consent being obtained, participants will complete a baseline survey and will be randomised into the KIND Challenge condition or a waitlist condition.

### Allocation and blinding

Simple randomization (1:1) will be used as the study is conducted entirely on online questionnaire software Qualtrics. Blinding is not possible in this RCT and there may be recruitment biases that may occur (e.g., those who choose to participate may already be more altruistic and more open to being engaged within research studies).

### Data collection and management

#### Data collection

Assessment data will be collected online via the Qualtrics survey platform using a digital case report form. Data will be extracted to a secure data file and stored on a secure network. Data is screened for duplicates and removed by the research assistant using categorisation function on Microsoft Excel. For each stage of data cleaning and analysis that is performed, a separate time-stamped computer file will be created and saved within an organised file system. To aid data quality, checks will include examination of recorded data for out-of-range values and data entry errors.

#### Data management

Data collected in this randomized control trial will be managed in accordance with the relevant research ethics board. All researchers will have to follow the relevant ethics procedures and protocols for managing and storing data. Restrictions to the data will be made such that only those listed as an author in this protocol, as well as the host institutes, will have access to the data. The host institutes access will be specific to audit and regulatory processes and will not be used for any purpose outside the scope of the trial registry.

### Statistical methods

All analyses will be conducted individually for each country to reflect differing responses to COVID-19 related social restrictions in place during the intervention. This means that there will be three separate randomised controlled studies. Initial screening will be conducted to check normality and score distribution (e.g., skew and kurtosis) on all outcome variables. Where data distributions fall outside of acceptable levels of normality, subsequent analysis will account for that using non-normality robust estimators (such as the Mplus estimator MLR; [57]). To ensure randomisation was successful, balance at baseline will be assessed between the trial and control group on all outcomes i.e., primary outcome loneliness, secondary outcome social isolation, and secondary neighbourhood relationship quality and contact outcomes (c) social cohesion and trust, (d) perception that their neighbourhood is improving, (e) importance around getting to know their neighbours, (f) supportive, aversive, or ambivalent neighbour network classification, (g) neighbourhood conflict, (h) number of neighbourhood contacts, and the secondary mental health outcomes, (i) depression, (j) social anxiety, (k) stress, (l) quality of life, and (m) positive affect. MANOVAs will be used for interval data and Chi square for nominal data; effect size differences will be reported.

To assess trial effects, ITT analysis will provide an unbiased estimate of the impact of the intervention [58] comparing participants assigned to the KIND Challenge with those in the control group. ITT includes every participant that is randomised and ignores adherence and compliance and anything that happens post randomisation. Thus, participants are analysed as randomised, reducing bias and with the purpose of ensuring groups differ only on the intervention being compared. ITT, therefore, represents real practical scenarios in which lack of adherence and compliance would naturally be encountered. ITT will be applied to H1–4. ITT, however, suffers from issues of non-compliance because participants assigned to the intervention may not engage with it and, thus, would bias and dilute impact of the intervention. Therefore, H7 aims to explore how compliance, adherence, and engagement with the KIND Challenge intervention moderate impact on outcomes; H8 will assess the impact of compliance on outcomes, using Complier Average Causal Effects (CACE).

### Outcomes analysis

An ITT analyses approach, using linear multiple regression modelling in Mplus 8.3, will be employed. A KIND Challenge intervention effect can be noted if the coefficients associated with the trial group variable are statistically significant and effect size recorded.

A model building approach will be used. Model 1 will include loneliness as the outcome variable, with explanatory co-variates gender, age group, baseline loneliness respectively. The target variable trial group (KIND Challenge versus control group) will be added with control group as the reference group (coded 0). Sensitivity analysis will be conducted to determine if effects are robust and are maintained once additional explanatory variables are controlled for.

Model 2 will build in additional secondary and mental health explanatory variables, e.g., baseline social isolation, depression, social anxiety, stress, quality of life, positive affect, given their known associations with the response variable.

Model 3 will build in additional community explanatory variables, e.g., baseline levels of Nextdoor platform activity (both active and passive use), baseline community engagement, Pre-KIND Challenge neighbourhood contact and previous kindness activity engagement, and neighbourhood relationship quality variables of (a) social cohesion and trust, (b) perception that their neighbourhood is improving, (c) higher importance around getting to know their neighbours, (d) feelings of ambivalence towards their neighbours, supportive, aversive, or ambivalent neighbour network classification, (e) neighbourhood conflict, and (f) number of neighbourhood contacts.

Model 4 will build in controlling for COVID-19 climate by asking respondents the number of government restrictions imposed (from a list of 13 types of restrictions).

### Secondary outcomes analysis

The above ITT analysis using linear multiple regression modelling in Mplus 8.3, will be repeated for the secondary outcome of social isolation (H2) and the neighbourhood relationship quality outcomes H3 (a) social cohesion and trust (measured using the Social Capital Scale) at post 4-week intervention. For outcomes (b) perception that their neighbourhood is improving (categorised at improving, stable or declining), and (d) supportive, aversive, or ambivalent neighbour network classification, because those are categorical data, we will use a multi-nominal regression. Outcomes (c) higher importance around getting to know their neighbours (high and low importance), (e) neighbourhood conflict (no neighbourhood conflict and instances of neighbourhood conflict), and (f) number of neighbourhood contacts (0–5 and 6+ neighbourhood contacts) are dichotomous categorical data so we will use a binominal regression to explore ITT effects. Similarly, for all mental health secondary outcomes at post 4-week intervention, H4 (a) depression (b) social anxiety, (c) stress, (d) quality of life, and (e) positive affect, measured using the PHQ-8, Mini-SPIN, PSS-4, EUROHIS-QOL-8, and PANAS-PA respectively. The same model building approach will be adopted as in H1.

To model whether effects are maintained for all outcomes (H5) in the participants assigned to the KIND Challenge group, a series of within-group ANCOVAs will be run. Differences in baseline (T1), post-intervention (T2), and one-month follow-up (T3) outcomes will be analysed for both primary and secondary outcomes. A model building approach will be taken controlling co-variates outlined above for H1–4. The sustainability of the intervention (H6) will be explored to determine if the participants assigned to the KIND Challenge group continue with acts of kindness. Both the frequency and duration of KIND acts completed in the one-month follow-up, and the level of engagement for each type of activity completed (i) emotional support, (ii) tangible support, (iii) informational support, (iv) belonging support, and (v) companionship support will be analysed. A series of within-group ANCOVAs will be run using the frequency and duration of KIND acts completed post 4-week intervention and one-month follow-up, to determine if engagement is sustained.

For feasibility, acceptability, and safety, we will present the descriptive statistics on these indicators (feasibility = < 40% drop out; acceptability more than 5 or higher on meaningful, safe, positive, and comfort ratings; and no reported conflicts because of doing the KIND challenge).

### Adherence and engagement variability

Variability in adherence and engagement will be measured in the participants allocated to the KIND Challenge group. First, adherence can be measured by the frequency and duration of KIND acts completed during the 4-week intervention. Second, an index of level of engagement for each type of activity completed (i) emotional support, (ii) tangible support, (iii) informational support, and (iv) belonging support, (v) companionship support can be created by combining the frequency and duration to produce an indicator of dosage for each type of KIND act. Third, the impact of overall engagement with the KIND Challenge can be modelled via meaningfulness, degree of positive feelings and sense of connectedness to others.

Linear multiple regression modelling in Mplus 8.3, will be employed to model the adherence and engagement as predictors of post 4-week intervention outcomes (H7), across the primary outcome of loneliness, and secondary outcome of social isolation, a neighbourhood relationship quality and contact, neighbourhood connectedness, and mental health. As with the H1–4 the model building approach will be used.

Reasons for non-compliance will also be captured and explored to assess barriers and challenges to competing the KIND activity, as measured by frequencies of lack of time, forgot, illness, work stress, family pressures or other.

CACE estimation will be employed using Mplus 8.3 to examine whether the effects noted in ITT analyses (H1–4) change once intervention compliance is taken into account. Participant demographics and baseline outcome variables will be used as predictors of the latent class compliance variable. Compliance will be assessed via dosage (i.e., number of kind acts completed; compliers vs. non-compliers). The CACE analysis will follow the same regression model building approaches detailed in H1–4.

### Additional analyses

The exploratory questions will be addressed to determine country differences on loneliness, social isolation, and levels of engagement with the KIND Challenge. MANOVAs and effect size differences will be reported. How loneliness and social isolation differ across neighbourhood characteristics (urban vs rural) and demographic variables (age, gender, socioeconomics) will also be explored. Two regression analyses will be run with loneliness and social isolation as respective outcomes and predictor variables of neighbourhood characteristics (urban or rural) and demographic variables of age, gender and socio-economic status added.

To account for the climate and context the data was collected in, how the COVID-19 pandemic has impacted loneliness and social isolation, and people’s interactions with their neighbours will be explored. Regression analyses will be run with baseline loneliness and social isolation, and baseline neighbourhood relationship quality outcomes: social cohesion and trust, perception that their neighbourhood is improving, importance around getting to know their neighbours, supportive, aversive, or ambivalent neighbour network classification, and neighbourhood conflict; and predictor variables of level of social restriction (i.e., social distancing, quarantine, social isolation, none applicable), number of government restrictions imposed (from a list of 13 types of restrictions), and the change in the amount of face-to-face interaction because of the COVID-18 pandemic (ranging from 1: interacting with almost no one face to face to 5: I have interacted with many more people face to face).

### Missing data

The proportion of missing data will be determined for a given outcome variable and the following procedures will be applied: i) if less than 5% of data are missing a complete case analysis will be undertaken, ii) if more than 5% and is missing and is missing at random (MAR), then multiple full information maximum likelihood (FIML) will be utilised [59, 60]. Post-trial, the extent and nature of missing data will be established, and differences between complete and missing cases will be examined to establish any pattern to the missingness [60]. Logistic regression will be used to predict missingness, whereby each participant will be coded as having complete (0) or incomplete (1) outcome data for loneliness and social isolation, with other study data as explanatory variables (e.g., trial group allocation, gender, age group, baseline scores on depression, social anxiety, QoL, positive affect, stress, neighbourhood relationship quality and contact variables). Where applicable FIML under the missing at random assumption will be utilised in Mplus 8.3. This will enable us to include both partially and completely observed cases of all participants in the analysis, thereby reducing the bias associated with attrition.

### Oversight and monitoring

#### Research governance and ethics

The trial is administered by Swinburne University of Technology. The study has been approved by Swinburne University Human Research Ethics Committee (SUHREC: 2020/2615), Brigham Young University Human Research Ethics Committee (IRB2020–175). The study received a waiver from University of Manchester as all identifiable data will be controlled and handled by Swinburne University of Technology. The trial is conducted in accordance with the Declaration of Helsinki, Good Clinical Practice guidelines, and the Australian National Statement on Ethical Conduct in Human Research [61]. Major protocol amendments will be submitted to SUHREC for review and approval and detailed in the trial registry. This project is subjected to the online audit tool, project progress review, and end of project review conducted yearly (or earlier if project closes before 12 months). The audit and reviews are conducted by the lead Chief Investigator. Full written informed consent is obtained from participants at the time of the baseline assessment by a member of the research team. A copy of the consent form can be obtained by requesting from the corresponding author.

There are no restrictions of the reporting findings of this trial. Nextdoor Inc., a commercial company, has not reviewed this protocol. Trial results will be published in full in the peer-reviewed literature. Media press releases and stakeholder reports will be released when minimum data is collected (baseline to end of Challenge). There is no intended use of professional writers to convey statistical data and findings.

#### Serious adverse events

An adverse event within this study is defined as participants reporting more than discomfort (e.g., distress) over a particular aspect of the current study. Due to the nature of the survey questions as well as the nature of the KIND Challenge (i.e., doing acts of kindness), we anticipate minimal risk to participants. Participants will be asked questions about their mental health but are not coerced into answering questions and can skip a question or close the survey at any time. Participants will be encouraged to seek counselling and support services if they feel they need someone to talk to. Participants will also have to the research team and ethics officer’s details should they choose to discuss any potential risk issue. The number for each country’s crisis service will also be provided in the participant information and consent form as an additional safety measure. If a participant opts for direct assistance and permits us to contact, we can debrief the participant and refer appropriate country specific services.

#### Trial management committee

A trial management committee will involve the named authors on this protocol as well as an independent academic not associated with the project. The project will be coordinated by the chief investigator who will also collaborate with senior co-investigators with regards to overseeing the trial. The chief investigator will ensure that each member of the trial management committee will be responsible for ensuring the safety of the participants and the quality of the trial is not compromised.

## Discussion

Loneliness is an emerging issue in our communities and while solutions to loneliness are often widely implemented, these are rarely evaluated [3]. One strategy that holds the potential to improve relationship quality is doing acts of kindness. The aim of this study is to assess the acceptability, feasibility, and effectiveness of an evidence-based strategy, specifically acts of kindness, on loneliness and social isolation. While positive psychology strategies have been thought to be useful in reducing loneliness, these strategies are not evaluated within our community. This study will be the first study of its kind to evaluate the impact of kindness on loneliness in communities across three countries. It is therefore crucial that we evaluate the effectiveness of these simple positive actions on loneliness and social isolation in our community.

An additional point to note is that this study is conducted during the COVID-19 pandemic. The COVID-19 pandemic has highlighted the social needs of vulnerable people in our community, and it is more important than ever to assist our community [62]. Loneliness and social isolation are consequences of this global public health crisis. Countries such as the UK, USA, and Australia who are participating in this research are undergoing different social restrictions which we have to statistically control for. Furthermore, acts of kindness typically conducted in person will have to be performed within safe and flexible ways. Participants should first and foremost adhere to their government or state health regulations to ensure safety of the participant and those who they interact with. This will be emphasised through the study. Despite the COVID-19 pandemic challenges posed to the study, there is still flexibility around how participants show acts of kindness safely and have given recommendations on how they can do them safely (e.g., calling a neighbour). Promoting community cohesion during this public health crisis is more crucial than ever and if effective, simple actions such as acts of kindness should be encourage in order to promote community cohesion, health and wellbeing.

### Trial status

Six months from 6 July 2020 to 31st January 2021.

## Supplementary Information

**Additional file 1: Supplementary Table 1.** Bespoke Study Questions.

## Data Availability

The datasets during the current study are available from the corresponding author on reasonable request and only by approval from the funder. Should data be released, all identifying data will be removed in order to adhere to confidentiality guidelines stipulated in this trial.

## Abbreviations

PPA: Positive psychology activities
RCT: Randomised controlled trial
UCLA-LS: UCLA Loneliness Scale - Version 3
LSNS: Lubben Social Network Scale
SRI: Social Relationship Index
PHQ: Patient Health Questionnaire-8
Mini-SPIN: Mini-Social Phobia Inventory
EUROHIS-QOL-8: European Health Interview Survey-Quality of Life – 8-Item Index
PANAS-PA: The Positive and Negative Affect Schedule – SF
PSS-4: Perceived Stress Scale-4
MDES: Minimum detectable effect size
ITT: Intention-to-treat
MAR: Missing at random
FIML: Full information maximum likelihood
SUHREC: Swinburne University Human Research Ethics Committee
