# Supplementary material for: A randomised controlled trial of the Nextdoor Kind Challenge: a study protocol
Source: BMC Public Health. 2021 Aug 5;21:1510. doi: 10.1186/s12889-021-11489-y (PMC8339389; doi:10.1186/s12889-021-11489-y)
Supplement: Supplementary file 1 — Additional file 1: Supplementary Table 1. Bespoke Study Questions. [file 12889_2021_11489_MOESM1_ESM.pdf]

Supplementary Table 1
Bespoke Study Questions

Questions	Item	Response scale
a) Neighbourhood conflict	In the past month, have you had any conflict with a neighbour?	1 = Yes<br>2 = No
	In the past month, have any of your neighbours made critical comments toward you?	1 = Yes<br>2 = No
	In the past month, have you been critical of others in your neighbourhood?	1 = Yes<br>2 = No
b) Number of contacts	How many people do you know in your neighbourhood?	1 = 0-5<br>2 = 6-10<br>3 = 11-15<br>4 = 15 +
c) Post KIND Challenge activity	Since receiving the KIND challenge, did you complete a Nextdoor KIND Challenge activity to connect with a neighbour?	1 = Yes<br>2 = No
	**If no**,<br>If you did not complete an activity, what factors stopped you from completing the activity?	1 = Lack of time<br>2 = Forgot<br>3 = Illness<br>4 = Work stress<br>5 = Family pressures<br>6 = Other, please specify
	**If yes**,<br>Which activity did you do? Select all that apply.	1 = Showed care and concern for a neighbour (e.g., provided a listening ear, cheered up a neighbour who was down, checked in on the welfare of a neighbour)<br>2 = Provided advice or helpful information to a neighbour (e.g., where to shop, good doctors in the

		area, potential job opportunities, gardening tips, etc.)<br>3 = Helped a neighbour out (e.g., mowed their lawn, took in their garbage bins, offered meals, ran errands, brought them groceries, etc.)<br>4 = Contributed to a larger neighbourhood effort, action, or activity (e.g., supported a neighbourhood business, shared your talents/skills with others, neighbourhood clean up, volunteering, etc)<br>5 = Had regular contact with a neighbour (e.g., chatted to a neighbour across the fence, street, or balcony, called a neighbour on the phone, etc)<br>6 = Other, please specify what you did:
	For each activity elected from above, a participant will be asked the following:<br><br>How often did you complete *insert activity name*?	1 = Once<br>2 = 1 – 3 times<br>3 = 3 – 5 times<br>4 = More than 5 times
	In total, how long did the activity take you?	1 = Less than 10 minutes<br>2 = Less than 30 minutes<br>3 = Less than 1 hour<br>4 = 1 hour to 2 hours<br>5 = More than 2 hours
	How connected did you feel to your neighbours because of the activity?	1 = Not at all connected to<br>10 = Very connected

	For the questions below, we ask that you reflect on your experience involved in the Nextdoor KIND Challenge.<br><br>How meaningful was the KIND Challenge to you?	1 = Not very meaningful to<br>10 = Very meaningful
	How safe did you feel when completing the KIND Challenge?	1 = Not very safe to<br>10 = Very safe
	How did you feel after doing the KIND Challenge?	1 = Not very positive to<br>10 = Very positive
	How connected did you feel to your neighbours because of the KIND Challenge?	1 = Not at all connected to<br>10 = Very connected
	How comfortable are you now in contacting your neighbours or others?	1 = Not at all comfortable to<br>10 = Very comfortable
d) Safety	Have you had a conflict with a neighbour **because** of the Nextdoor KIND Challenge?	1 = Yes<br>2 = No
e) Post KIND Challenge overall impact	Please rate the extent to which you agree with the following statements.<br><br>**After the Nextdoor KIND Challenge...**<br>I looked forward to being with my neighbours	1 = Extremely Disagree<br>2 = Disagree<br>3 = Neutral<br>4 = Agree<br>5 = Extremely Agree
	I looked forward to being with my neighbours	1 = Extremely Disagree<br>2 = Disagree<br>3 = Neutral<br>4 = Agree<br>5 = Extremely Agree
	I felt more connected with my neighbours	1 = Extremely Disagree<br>2 = Disagree<br>3 = Neutral<br>4 = Agree<br>5 = Extremely Agree
	I felt more socially confident	1 = Extremely Disagree

		2 = Disagree<br>3 = Neutral<br>4 = Agree<br>5 = Extremely Agree
	I formed new friendships and/or relationships with my neighbours	1 = Extremely Disagree<br>2 = Disagree<br>3 = Neutral<br>4 = Agree<br>5 = Extremely Agree
	I felt more connected to my community	1 = Extremely Disagree<br>2 = Disagree<br>3 = Neutral<br>4 = Agree<br>5 = Extremely Agree
	I felt more positive	1 = Extremely Disagree<br>2 = Disagree<br>3 = Neutral<br>4 = Agree<br>5 = Extremely Agree
	What can help you feel more connected to others?<br>Write in text box	Textbox entry
	What does being connected to people around you mean to you?<br>Write in text box	Textbox entry
	Would you change anything about the way the Nextdoor KIND Challenge was ran? If yes, please explain below.	Textbox entry
	Were there things that stopped you from connecting with others?	Textbox entry
f) Follow up KIND Challenge activity	**In the past month**, have you been in contact with your neighbour(s) either safely in person, or via a digital communication device?	1 = Yes<br>2 = No
	**If yes,**<br>Did you do any of the following? Select all that apply	1 = Showed care and concern for a neighbour (e.g., provided a listening

		ear, cheered up a neighbour who was down, checked in on the welfare of a neighbour)<br>2 = Provided advice or helpful information to a neighbour (e.g., where to shop, good doctors in the area, potential job opportunities, gardening tips, etc.)<br>3 = Helped a neighbour out (e.g., mowed their lawn, took in their garbage bins, offered meals, ran errands, brought them groceries, etc.)<br>4 = Contributed to a larger neighbourhood effort, action, or activity (e.g., supported a neighbourhood business, shared your talents/skills with others, neighbourhood clean up, volunteering, etc)<br>5 = Had regular contact with a neighbour (e.g., chatted to a neighbour across the fence, street or balcony, called a neighbour on the phone, etc)<br>6 = Other, please specify what you did:
	**If yes,**<br>**In the past month**, how often did you complete one of these activities?	1 = Once<br>2 = 1 – 3 times<br>3 = 3 – 5 times<br>4 = More than 5 times
	**If yes,**	1 = Less than 10 minutes

	**In total**, how long did the activity or activities take you?	2 = Less than 30 minutes<br>3 = Less than 1 hour<br>4 = 1 hour to 2 hours<br>5 = More than 2 hours
	**If yes,**<br>How connected did you feel to your neighbours because of the activity/activities?	1 = Not at all connected to<br>10 = Very connected
	Have you continued the Nextdoor KIND Challenge activities?	1 = Yes<br>2 = No
	**If yes,**<br>Did you do any of the following? Select all that apply	1 = Showed care and concern for a neighbour (e.g., provided a listening ear, cheered up a neighbour who was down, checked in on the welfare of a neighbour)<br>2 = Provided advice or helpful information to a neighbour (e.g., where to shop, good doctors in the area, potential job opportunities, gardening tips, etc.)<br>3 = Helped a neighbour out (e.g., mowed their lawn, took in their garbage bins, offered meals, ran errands, brought them groceries, etc.)<br>4 = Contributed to a larger neighbourhood effort, action, or activity (e.g., supported a neighbourhood business, shared your talents/skills with others, neighbourhood clean up, volunteering, etc)

		5 = Had regular contact with a neighbour (e.g., chatted to a neighbour across the fence, street or balcony, called a neighbour on the phone, etc)<br>6 = Other, please specify what you did:
	**If no,**<br>What were the main reasons for not continuing the Nextdoor KIND Challenge activities? Please answer in the textbox below:	Textbox entry
g) Nextdoor platform activity	How long have you been a Nextdoor user?	1 = Less than 1 week<br>2 = Less than 1 month<br>3 = Less than 6 months<br>4 = A year<br>5 = Between 1-5 years<br>6 = More than 5 years
	**In the past month,** can you estimate the amount of time you spent actively contributing to the Nextdoor platform? This may include interacting with other users via private message, responding to events in your neighbourhood, or contributing to other social media posts from your neighbours.	1 = Less than 1 hour<br>2 = 1 – 4 hours<br>3 = 5 – 9 hours<br>4 = More than 9 hours
	What functions of the Nextdoor website and/or app do you engage with regularly? Please select all that apply.	1 = Help me<br>2 = Buy and sell<br>3 = Events<br>4 = Crime and safety<br>5 = Lost and found<br>6 = Business<br>7 = Group pages<br>8 = Other<br>9 = None of the above

	**In the past month**, how many times have you posted on the Nextdoor website?	1 = Have not posted<br>2 = Once<br>3 = Twice<br>4 = 3 – 4<br>5 = 5-8<br>6 = 9+
	**In the past month,** can you estimate the amount of time you spent reading Nextdoor posts or browsing your Nextdoor newsfeed?	1 = Less than 1 hour<br>2 = 1 – 4 hours<br>3 = 5 – 9 hours<br>4 = More than 9 hours
	**Since the COVID-19 virus was declared a global pandemic on the 11th March 2020 by World Health Organisation...**<br><br>Has there been a change to the way you use the Nextdoor website and/or app?	1 = Yes<br>2 = No
	**If yes,**<br>How has it affected your time spent on the Nextdoor website and/or app?	1 = I have been spending **more** time on Nextdoor<br>2 = I have been spending **less** time on Nextdoor<br>3 = I have spent no more or less time on Nextdoor
	**If more time selected,**<br>For what reasons have you been using the Nextdoor website and/or app more? Select all that apply	1 = For updated information specified to my neighbourhood<br>2 = To offer help to my neighbours during this time<br>3 = To receive help from my neighbours who have offered<br>4 = To stay connected to my neighbours safely

		5 = To fill in time<br>6 = My reasons for using Nextdoor have not changed<br>7 = Other; please specify;
	**If less time selected,**<br>Why have you been spending less time on the Nextdoor website and/or app?	Text box entry
h) Neighbour interactions since COVID-19	**Since the COVID-19 pandemic began**, has there been a change to the overall frequency of contact you have had with your neighbours (either in person or via digital communication)?	1 = There has been no change<br>2 = I have been in contact with my neighbours **more**<br>3 = I have been in contact with my neighbours **less**
	**If more,**<br>In what ways have you been in contact with your neighbours? Select all that apply	1 = In person (no restriction)<br>2 = In person but at a safe distance away (at least 2 metres/ 6 feet away)<br>3 = Via phone, text, videocall or other communication device<br>4 = Other; please specify
	**If more,**<br>Is the type of contact (e.g., in person, phone, online) different to how you communicated with your neighbour(s) before the COVID-19 pandemic?	1 = Yes<br>2 = No
	**If less,**<br>why have you been in contact with your neighbours less?	Text box entry
	**For the questions below, please consider how you feel, act, or behave currently in response to the COVID-19 pandemic and answer accordingly.**<br>I worry about **my** health if I interact with my neighbour(s) in person	1 = I don't worry about this at all to<br>10 = I worry about this a lot
	I worry about **other's** health if I interact with my neighbour(s) in person	1 = I don't worry about this at all to<br>10 = I worry about this a lot
	I have avoided interacting with my neighbour(s) in person	1 = I have not done this at all to

		10 = I have done this a lot
	I have begun to use other methods of communication to get in contact with my neighbour(s)	1 = I strongly disagree to<br>10 = I strongly agree
	I worry the quality of the relationship I have with my neighbour(s) will become less meaningful	1 = I don't worry about this at all to<br>10 = I worry about this a lot
i) COVID-19 social restrictions	At the time of completing this survey, which of the following government policies/ precautions have been implemented in your country/community?<br><br>*Please select all that apply.*	1 = Closure of recreation centres, gyms, shopping centres, restaurants etc.<br>2 = Restriction of outdoor gatherings over a certain size<br>3 = Restriction of indoor gatherings over a certain size<br>4 = Travel ban (International)<br>5 = Travel ban (Domestic)<br>6 = Border closures at a state level<br>7 = Border closures at a national level<br>8 = Compulsory quarantine for anyone who has travelled or close contact of someone with COVID-19<br>9 = Compulsory quarantine for entire population<br>10 = Restrictions on number of essential items that can be bought from supermarkets/pharmacies<br>11 = University closures<br>12 = School closures<br>13 = Imposed restrictions on when and for what reasons to leave the home/place of residence
j) Pre-KIND Challenge	**In the past month,** have you been in contact with your neighbour(s) either safely in person, or via a digital communication device?	1 = Yes<br>2 = No

neighbourhood contact and previous kindness activity	**If yes,**<br><br>Did you do any of the following? Select all that apply.	1 = Showed care and concern for a neighbour (e.g., provided a listening ear, cheered up a neighbour who was down, checked in on the welfare of a neighbour)<br>2 = Provided advice or helpful information to a neighbour (e.g., where to shop, good doctors in the area, potential job opportunities, gardening tips, etc.)<br>3 = Helped a neighbour out (e.g., mowed their lawn, took in their garbage bins, offered meals, ran errands, brought them groceries, etc.)<br>4 = Contributed to a<br>larger neighbourhood effort, action, or activity (e.g., supported a neighbourhood business, shared your talents/skills with others, neighbourhood clean up, volunteering, etc)<br>5 = Had regular contact with a neighbour (e.g., chatted to a neighbour across the fence, street or balcony, called a neighbour on the phone, etc)<br>6 = Other, please specify what you did:
	**If yes,**<br>**In the past month**, how often did you complete one of these activities?	1 = Once<br>2 = 1 – 3 times<br>3 = 3 – 5 times

		4 = More than 5 times
	**If yes,**<br>In total, how long did the activity or activities take you?	1 = Less than 10 minutes<br>2 = Less than 30 minutes<br>3 = Less than 1 hour<br>4 = 1 hour to 2 hours<br>5 = More than 2 hours
	**If yes,**<br>How connected did you feel to your neighbours because of the activity/activities?	1 = Not at all connected to<br>10 = Very connected
